# Posttranslational Modifications: Regulation of Nitrogen Utilization and Signaling

**DOI:** 10.1093/pcp/pcab008

**Published:** 2021-01-25

**Authors:** Wei Wang, Aifu Li, Zhihua Zhang, Chengcai Chu

**Affiliations:** State Key Laboratory of Plant Genomics, Institute of Genetics and Developmental Biology, The Innovative Academy for Seed Design, Chinese Academy of Sciences, Beijing 100101, China; State Key Laboratory of Plant Genomics, Institute of Genetics and Developmental Biology, The Innovative Academy for Seed Design, Chinese Academy of Sciences, Beijing 100101, China; State Key Laboratory of Plant Genomics, Institute of Genetics and Developmental Biology, The Innovative Academy for Seed Design, Chinese Academy of Sciences, Beijing 100101, China; School of Life Sciences, Guangzhou University, Guangzhou 510006, China; State Key Laboratory of Plant Genomics, Institute of Genetics and Developmental Biology, The Innovative Academy for Seed Design, Chinese Academy of Sciences, Beijing 100101, China

**Keywords:** posttranslational modification, nitrogen utilization, nitrate signaling, phosphorylation, ubiquitination, sumoylation

## Abstract

Nitrogen is the most important macroelement required for the composition of key molecules, such as nucleic acids, proteins and other organic compounds. As sessile organisms, plants have evolved sophisticated mechanisms to acquire nitrogen for their normal growth and development. Besides the transcriptional and translational regulation of nitrogen uptake, assimilation, remobilization and signal transduction, posttranslational modifications (PTMs) are shown to participate in these processes in plants. In addition to alterations in protein abundance, PTMs may dramatically increase the complexity of the proteome without the concomitant changes in gene transcription and have emerged as an important type of protein regulation in terms of protein function, subcellular localization and protein activity and stability. Herein, we briefly summarize recent advances on the posttranslational regulation of nitrogen uptake, assimilation, remobilization and nitrogen signaling and discuss the underlying mechanisms of PTMs as well as the signal output of such PTMs. Understanding these regulation mechanisms will provide novel insights for improving the nitrogen use efficiency of plants.

## Introduction

Nitrogen is an essential macroelement for the composition of most biomacromolecules and many secondary and signaling compounds in plants, such as proteins, nucleic acids, cell wall components, phytohormones and vitamins (Krapp 2015, Zhang et al. 2020). The deficiency of nitrogen could severely limit plant growth and development. Except for few species, such as legumes, which can fix atmospheric N_2_ for nitrogen assimilation and metabolism, most plants mainly use nitrate in aerobic soils whereas ammonium in flooded wetland or acidic soils (Xuan et al. 2017). Due to the low availability of inorganic nitrogen in natural environments, plants have evolved sophisticated mechanisms to survive under temporal and spatial nitrogen fluctuations. Dissecting the molecular mechanisms involved in nitrogen uptake, assimilation, translocation, recycling and remobilization as well as nitrogen signaling network would pave the way for improvement of nitrogen use efficiency (NUE) and therefore decrease the input of nitrogen fertilizers so as to avoid environmental pollution (Xu et al. 2012, Wang et al. 2018).

Posttranslational modifications (PTMs) refer to various changes occurred at the polypeptide chain of proteins, including alterations by proteolytic cleavage, formation of disulfide bonds or covalent attachment of phosphate, sulfate, alkyl groups, lipids, carbohydrates, polypeptides and others (Aslebagh et al. 2019). It is now estimated that there are about 200 types of PTMs and 300 types of chemical and biological modifications presented in the UniProt database (https://sparql.uniprot.org/) (Bateman et al. 2019, Soudy et al. 2020). Distinct PTMs might have critical roles in dynamically regulating physical and chemical properties, folding, conformation distribution, stability, activity and consequently functions of the proteins and therefore promoting a majority of cellular processes. Recently, more and more reports have pointed out that many critical proteins responsible for nitrogen utilization and signal transduction are strongly regulated by PTMs, including phosphorylation, ubiquitination and sumoylation, and PTMs are considered to be of great significance for rapid response to sudden changes in nitrogen availability.

Protein phosphorylation is the most well-investigated reversible protein modification, which represents 53.5% of the PTMs based on the published data (Silva-Sanchez et al. 2015). Phosphorylation is catalyzed by protein kinases, and dephosphorylation is mediated by protein phosphatases. In Arabidopsis, protein phosphorylation mainly occurs at the hydroxyl groups of the hydroxyl amino acids serine (Ser, 80–85%), threonine (Thr, 10–15%) and tyrosine (Tyr, 0–5%) (van Wijk et al. 2014, Millar et al. 2019). Phosphorylation of cell membrane receptors and transporters in plants usually regulates their activity and protein stability and thus affects diverse signaling pathways in which the phosphorylated protein is involved. Different from protein phosphorylation that is modified by a single phosphate group, protein ubiquitination usually involves the attachment of a single ubiquitin molecule (mono-ubiquitination) or a chain of ubiquitin molecules (poly-ubiquitination) to lysine (K) residues of the target protein (Vierstra 2012). Ubiquitin is a small (76-amino-acid), stable, highly conserved protein usually linked to the target protein by the sequential participation of three proteins: ubiquitin-activating enzyme (E1), ubiquitin-conjugating enzyme (E2) and ubiquitin protein ligase (E3). Ubiquitination plays an important role in protein turnover, cellular signaling, intracellular localization, protein–protein interaction, transcriptional regulation and most notably in targeting proteins to the 26S proteasome or the lytic vacuole for degradation (Schwechheimer et al. 2009). Similar to ubiquitination, sumoylation, the covalent attachment of the small ubiquitin-like modifier protein (SUMO, approximately 110 amino acids) to the substrate proteins, provides an essential regulatory mechanism to control the protein activity, subcellular localization and fate of many intracellular processes in eukaryotes, but not typically targets proteins to degradation (Geiss-Friedlander and Melchior 2007, Wilkinson and Henley 2010). Furthermore, sumoylation rapidly rises upon stress, including nutrient limitation, and modulates responses for many environmental challenges (Miura et al. 2007, Miura and Hasegawa 2010, Elrouby 2017).

In this review, we mainly focus on the recent advances in the PTM regulations of nitrogen uptake, assimilation, remobilization and nitrogen signaling, emphasize the molecular mechanisms of PTMs and discuss the problems involved in crosstalk of PTMs in nitrogen signaling network in the future.

## Posttranslational Regulation of Nitrogen Uptake and Remobilization

### Role of phosphorylation in regulating ammonium uptake

**Fig. 1:**
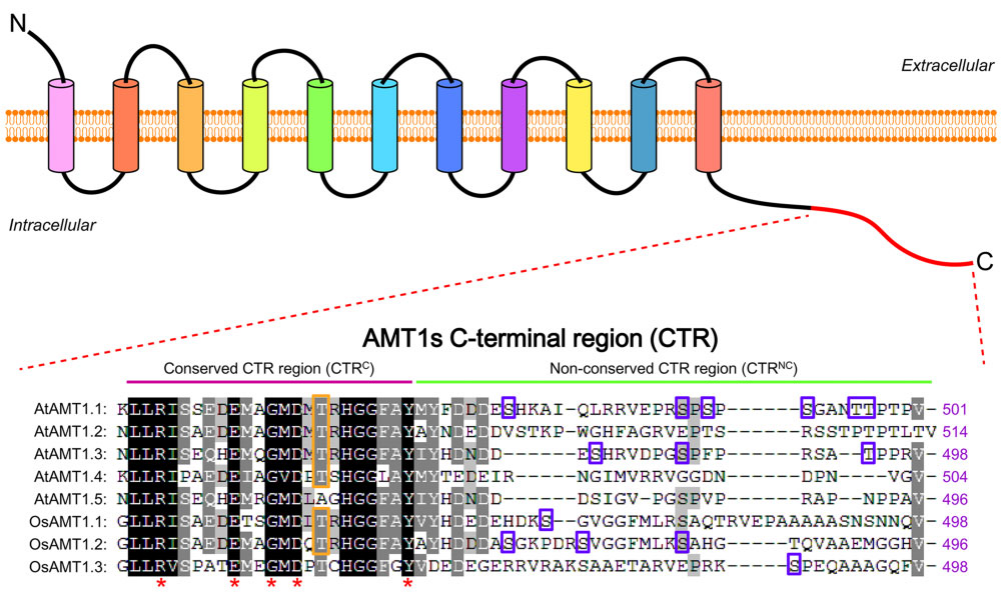
Phosphorylation sites in the CTR of AMT1s identified by in vivo phosphoproteomic approaches in Arabidopsis and rice, respectively (https://www.psb.ugent.be/webtools/ptm-viewer/resources.php) (Willems et al. 2019). The upper part of graph is the schematic diagram of transmembrane topology for AMT1s. Protein sequence alignment showed that the CTR in AMT1s consists of a conserved region (CTR^C^) and a non-conserved region (CTR^NC^) underlined with purple and green, respectively. The accession numbers of the AMT1 proteins from Arabidopsis and rice are as follows: AtAMT1.1 (AT4G13510), AtAMT1.2 (AT1G64780), AtAMT1.3 (AT3G24300), AtAMT1.4 (AT4G28700), AtAMT1.5 (AT3G24290), OsAMT1.1 (LOC_Os04g43070), OsAMT1.2 (LOC_Os02g40730) and OsAMT1.3 (LOC_Os02g40710). At, *Arabidopsis thaliana*; Os, *Oryza sativa*. The phosphorylated Thr residue in the CTR^C^ identified among all accessions except for OsAMT1.3 are labeled with orange boxes. Other phosphorylated Ser/Thr residues in the CTR^NC^ are labeling with cyan boxes. Asterisks indicate the conserved residues within the CTR^C^, including the ‘ExxGxD’ motif.

For most plants, ammonium transporters (AMTs) and nitrate transporters are two major kinds of transporters that were responsible for inorganic nitrogen uptake and translocation. Most AMTs belong to the AMT/Methylammonium Permease/Rhesus family with the properties of high affinity and selectivity, and saturation at ammonium concentration <1 mM (Nacry et al. 2013). Phosphorylation of AMTs was well characterized, revealing an important mechanism of posttranslational regulation for the control of ammonium transport activity. Phosphorylation of AtAMTs was first identified in a phosphoproteomic screen for membrane proteins, in which AtAMT1.1 was found to be phosphorylated at Thr460 with the supplement of 20 mM NH_4_NO_3_ (Nuhse et al. 2004). Further study revealed that phosphomimic variant AtAMT1.1^T460D^ via substitution of threonine with aspartate leads to an inactive AMT, while the dephosphomimic variant AtAMT1.1^T460A^ via substitution of threonine with alanine results in an active AMT (Loque et al. 2007). This Thr460 residue at the carboxyl-terminal region (CTR) of AtAMT1.1 is equivalent to Thr472 in AtAMT1.2 and Thr464 in AtAMT1.3, and phosphorylation of the relevant threonine site results in a rapid shut-off mechanism to avoid ${\text{NH}}_4^ + $ toxicity under high ammonium concentrations, suggesting that the phosphorylation mode may be of universal significance among AMT1s (Fig. 1) (Neuhauser et al. 2007, Lanquar et al. 2009, Yuan et al. 2013, Li et al. 2014).

However, the potential regulatory mechanisms of phosphorylation/dephosphorylation of AMTs exerted by protein kinases/phosphatases were not entirely clear. In 2017, the protein kinase CIPK23 (Calcineurin B-Like Interacting Protein Kinase 23) was first shown to phosphorylate the T460 residue in AtAMT1.1 and the T472 residue in AtAMT1.2 in a CBL1 (Calcineurin B-Like protein 1)-dependent manner (Straub et al. 2017). Nevertheless, phosphorylation signals of the relevant conserved threonine residues in AtAMT1.1 and AtAMT1.2 were still detectable in *cipk23* mutant by using phospho-specific antibody, even though the signal was reduced significantly, suggesting that additional protein kinases might participate in phosphorylation process besides CIPK23 (Straub et al. 2017). Soon afterward, OsACTPK1, another protein kinase belonging to the serine/threonine/tyrosine protein kinase family, was reported to regulate the T452 phosphorylation in OsAMT1.2 (relevant to T460 in AtAMT1.1) upon the increasing ammonium supply. Interestingly, OsACTPK1-mediated modulation of AMT1.2 largely relies on the transcriptional control of *OsACTPK1*. Under the condition of ammonium deficiency, *OsACTPK1* transcription is quite poor; thus, OsAMT1.2 is dephosphorylated and activated to acquire more ammonium from soil. Upon the condition of sufficient ammonium, the transcript level of *OsACTPK1* is rapidly increased, and the accumulation of OsACTPK1 protein leads to phosphorylation and inactivation of OsAMT1.2 to prevent ammonium toxicity (Beier et al. 2018). Moreover, phosphorylation of T464 in AtAMT1.3 was also detected in vivo using mass spectrometry-based proteomics, and the transition of AtAMT1.3 phosphorylation status is correlated with its transporter activity (Yuan et al. 2013, Menz et al. 2016). But the T464 residue in AtAMT1.3 is not the target of CIPK23, even though adjacent sequences of the phosphorylation site has no significant difference among AMTs (Straub et al. 2017).

In addition, treatment with either FK506 (an inhibitor of calcium-regulated serine/threonine-specific protein phosphatase) or genistein (a specific inhibitor of tyrosine-specific protein kinases) caused significant reduction in OsAMT1.1-mediated ammonium uptake at least by 30% in rice (Yang et al. 2015). And also, in addition to the conserved threonine in the CTR, other phosphorylation sites were identified in vivo by mass spectrometry, including six phospho-sites (S475, S488, S490, S492, T496, T497) in AtAMT1.1 and three phospho-sites (S480, S487, T494) in AtAMT1.3 (Fig. 1) (Reiland et al. 2009, Engelsberger and Schulze 2012, Menz et al. 2016). Collectively, these results suggested that AMTs might be modulated by dynamic phosphorylation/dephosphorylation modes within multiple phospho-sites in response to alterations of the nitrogen status, including different forms of nitrogen sources. Indeed, it was revealed that resupply of nitrate, but not ammonium, could trigger a fast dephosphorylation of T494 in the CTR of AtAMT1.3, which results in enhanced transport activity of AtAMT1.3, and thus increases the overall ammonium uptake in Arabidopsis (Wu et al. 2019). However, the phosphatase associated with the dephosphorylation of the T494 site in AtAMT1.3 remains unknown. Unlike CIPK23 negatively regulating transport activity of AtAMT1.1, CPK32 (Calcium-dependent Protein Kinase 32), another protein kinase recently identified through methylammonium (MeA) toxicity screening was found as a positive regulator involved in ammonium uptake (Qin et al. 2020). Actually, ammonium is typically the preferred nitrogen source for plants, and its concentration in most agricultural soils is in the range of 20–200 μM that is not enough to be toxic (Esteban et al. 2016). CPK32 could interact with AtAMT1.1 and phosphorylate AtAMT1.1 at the non-conserved serine residue Ser450 in the CTR to enhance ammonium uptake in the natural environment (Qin et al. 2020). This study revealed a new mechanism underlying regulation of normal ammonium uptake. Taken together, the posttranslational regulation of AMTs may involve phosphorylation/dephosphorylation events within multiple phospho-sites, which were triggered by complicated nitrogen signal input, further suggesting the complexity and diversity of ammonium uptake.

### Role of phosphorylation in regulating nitrate transporter activities

**Fig. 2:**
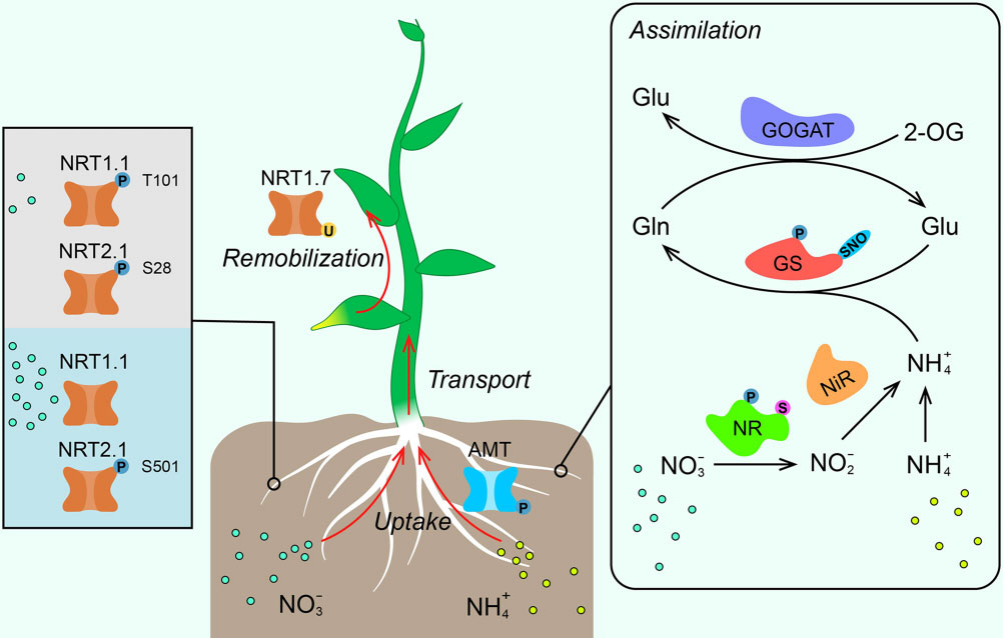
Illustration of the PTMs for the key components involved in nitrogen metabolism identified so far. NRTs are responsible for nitrate uptake, transport and remobilization, while AMTs are involved in ammonium uptake and transport. Detail functions and PTMs of three NRTs are indicated. For nitrogen assimilation, nitrate is converted to ammonium by NR and NiR, and then ammonium converted from nitrate, as well as ammonia taken directly from soil, is assimilated to different amino acids via GS/GOGAT cycle. All the PTMs are indicated with the corresponding chemical modification groups or proteins. P, phosphoryl group; U, ubiquitin; S, SUMO; SNO, *S*-nitrosothiol.

Currently, physiological functions of most nitrate transporters are well investigated using Arabidopsis as a model system, and two gene families have been identified responsible for nitrate uptake and transport: NPF (NRT1/PTR FAMILY, 53 members) and NRT2 (7 members). Among them, two well-studied genes, *AtNRT1.1* (*AtNPF6.3*) and *AtNRT2.1*, encoding a dual- and high-affinity nitrate transporter, respectively, have been revealed important regulatory mechanisms involving PTMs. Notably, the switch of AtNRT1.1 between the high- and low-affinity nitrate transport systems in response to environmental nitrate fluctuations is governed by the phosphorylation/dephosphorylation of Thr101 in AtNRT1.1. In response to low-nitrate availability, the Thr101 in AtNRT1.1 is phosphorylated by CIPK23, and AtNRT1.1 functions as a high-affinity nitrate transporter; upon high nitrate supply, it is dephosphorylated by an unknown phosphatase and works as a low-affinity nitrate transporter (Fig. 2) (Liu and Tsay 2003, Ho et al. 2009). Crystal studies further revealed that T101 phosphorylation affects the dimerization of AtNRT1.1. When nitrate is abundant, dephosphorylated AtNRT1.1 forms a dimer to decrease structural flexibility. Conversely, upon low-nitrate availability, phosphorylation of AtNRT1.1 at T101 decouples the homo-dimer, resulting in enhanced structural flexibility (Parker and Newstead 2014, Sun et al. 2014).

Given that the uptake of nitrate and ammonium is well balanced, and CIPK23 may simultaneously regulate the root uptake of nitrate and ammonium, it could be postulated that CIPK23 might be a key downstream component to maintain the balance of nitrate and ammonium in response to environmental nitrogen fluctuation (Straub et al. 2017). Upon moderate ammonium conditions and/or high nitrogen demand, CIPK23 is inactive and bound in the cytoplasm, and high nitrate uptake by nonphosphorylated AtNRT1.1 is balanced with ammonium uptake for ion charge balance by nonphosphorylated and active AMT1s. While under excessive ammonium and/or low nitrogen demand, CIPK23 is activated and recruited to the plasma membrane and phosphorylates AtNRT1.1 and AMT1s. Thereby, the nitrogen demand is met by high-affinity AtNRT1.1 and inactive AMT1s. In this context, CIPK23 might be of general interest and take part in the integration of different nitrogen signaling pathways to co-ordinate the utilization of nitrate and ammonium. However, there is no evidence that CIPK23 targeting is regulated by nitrogen status, and how CIPK23 responses to nitrogen status and fine-tunes nitrate and ammonium uptake remain to be answered.

For AtNRT2.1, three phosphorylation sites were identified in vivo by shotgun phosphoproteomics, including Ser11, Ser28 and Thr521 (Engelsberger and Schulze 2012, Menz et al. 2016). Ser28 of AtNRT2.1 was shown to be rapidly dephosphorylated in 10 min upon resupply of high concentrations of nitrate (3 and 10 mM KNO_3_) but remained phosphorylated under low concentration of nitrate (0.3 mM KNO_3_) (Engelsberger and Schulze 2012). This result suggested that dephosphorylation of AtNRT2.1 is important for regulating root nitrate uptake in response to high nitrate supply (Fig. 2). Subsequent study revealed that phosphorylation of AtNRT2.1 at Ser28 could stabilize the protein itself in response to nitrate limitation, and dephosphomimic AtNRT2.1^S28A^ failed to rescue the growth-restricted phenotype of the *atnrt2.1* mutant under low-nitrate supply (Zou et al. 2020). Another study has also shown that the Ser501 of AtNRT2.1 is a key phospho-site for AtNRT2.1 activity. Phosphorylation of Ser501 in AtNRT2.1 was found to be increased by ammonium nitrate (10 mM), resulting in the inactivation of AtNRT2.1 and the decrease in high-affinity nitrate uptake in roots (Fig. 2) (Jacquot et al. 2020). These results collectively showed that AtNRT2.1 has opposite phosphorylation/dephosphorylation patterns within N- and C-terminal regions, which are corresponding to AtNRT2.1 activity in response to varied environmental cues. However, the detailed regulatory mechanisms underlying AtNRT2.1 phosphorylation/dephosphorylation patterns and protein stability remain largely unknown.

### Role of ubiquitination in regulating nitrogen remobilization

In addition to nitrogen uptake, nitrogen remobilization is important in improving nitrogen use efficiency (NUE) and crop yield. Nitrogen remobilization is usually in the form of nitrate and amino acids (Hirel et al. 2001, Hortensteiner and Feller 2002), and nitrate remobilization among various organs is mainly mediated by nitrate transporters. Previous study has revealed that AtNRT1.7 (AtNPF2.13) located in the phloem is responsible for source-to-sink remobilization of leaf nitrate during nitrogen starvation (Fan et al. 2009). Due to the crucial function of AtNRT1.7 under nitrogen limitation, plants employ a regulatory mechanism to ensure the abundance of AtNRT1.7 with the circuit of the microRNA miR827 and NITROGEN LIMITATION ADAPTATION (NLA) (Liu et al. 2017b). AtNRT1.7 could interact with NLA, a RING-type E3 ligase, in the plasma membrane, and then was ubiquitinated by NLA for degradation via 26S proteasome pathway. Notably, NLA amount is translationally repressed by miR827 upon nitrogen deficiency (Fig. 2) (Liu et al. 2017b). Together, the protein abundance of AtNRT1.7 is increased due to the miR827-mediated reduction of NLA under nitrogen limitation so that nitrate remobilization from source to sink via phloem is enhanced to ensure the plant growth. Therefore, ubiquitination-mediated posttranslational regulatory plays an important role in regulating nitrate remobilization during nitrogen limitation.

## Posttranslational Regulation in Nitrogen Assimilation

Nitrogen assimilation in plants involves several key steps. Nitrate acquired from soil is first reduced by cytosolic nitrate reductase (NR or NIA) to nitrite, which was then imported into the chloroplasts or plastids for further converting to ammonium by nitrite reductase (NiR) (Krapp 2015, Zhang and Chu 2020). The ammonium converted from nitrate as well as ammonium taken up directly from soil is assimilated via the glutamine synthetase (GS)/glutamine oxoglutarate aminotransferase (GS/GOGAT) cycle (Fig. 2) (Krapp 2015, Zhang and Chu 2020). In addition, ammonium generated via photorespiration and protein degradation pathways is captured to reassimilate via the GS/GOGAT cycle (Xu et al. 2012). The activity of these assimilatory enzymes might be regulated at a number of different levels, especially for posttranslational regulation of their activities.

As a rate-limiting step for producing ammonium from nitrate, nitrate reductase activity is regulated partly by PTMs in accordance with internal (cellular) and external (environmental) cues (Fig. 2). In spinach, it was first shown that NR is phosphorylated at the Ser543 in the hinge 1 region, which leads to formation of a binding site for 14–3–3 proteins (R–XX–pS/pT–X–P), resulting in its inactivation (Bachmann et al. 1996). Subsequent study also identified a CDPK with closest sequence similarity to the Arabidopsis CDPK6 (also known as CPK3), which could phosphorylate NR at Ser543 to inhibit the NR activity in spinach (Douglas et al. 1998). By contrast, protein phosphatase 2A (PP2A) could interact with NR and thus activates its nitrate reduction activity (Heidari et al. 2011). However, whether PP2A could dephosphorylate NR and the relevant dephosphorylation site were not investigated. In addition, AtNIA2 was reported to physically interact with Mitogen-activated Protein Kinase 6 (MPK6), resulting in the phosphorylation at Ser627 in AtNIA2 and increased NR activity (Wang et al. 2010).

Interestingly, NR activity is also regulated by sumoylation. It is shown that the nitrate reductases in Arabidopsis, AtNIA1 and AtNIA2, are sumoylated by the E3 SUMO ligase AtSIZ1 both in vivo and in vitro, which significantly increased their enzyme activities and promoted nitrate assimilation in Arabidopsis (Park et al. 2011). In addition, the growth defect of *atsiz1* mutant is restored with exogenous ammonium but not nitrate, indicating that *atsiz1* mutant has a severe defect on nitrate assimilation (Park et al. 2011). Overall, sumoylation mediated by AtSIZ1 has an essential role in plant development through its effect on nitrate assimilation and, together with phosphorylation, is therefore a critical protein modification for regulating NR activity.

GS is also considered to be one of the key enzymes participated in nitrogen metabolism. It mediated the synthesis of glutamine from glutamate and ammonia using ATP as the energy donor. Although the transcriptional regulation of *GS* has been widely studied (Thomsen et al. 2014), and it is generally considered as the primary regulatory point mediated by upstream and downstream N and C metabolites, a number of posttranslational regulatory mechanisms were shown to be critical for the regulation of GS activity (Fig. 2), including phosphorylation (Finnemann and Schjoerring 2000, Lima et al. 2006), oxidative turnover (Choi et al. 1999), tyrosine nitration (Melo et al. 2011), and S-nitrosation (Silva et al. 2019). However, it remains elusive to get a comprehensive understanding of the posttranslational regulatory mechanisms of GS in plants. There are many GS isoforms with similar protein molecular weight and isoelectric point concurrently expressing in the same organs or tissues, and one GS isoform may have undergone multiple PTMs at the same time. Therefore, it is difficult to distinguish the effect of a given PTM on the individual GS isoform. Although many studies investigated the effect of a particular PTM on GS activity using in vitro recombinant enzymes, the biological significance of PTMs still requires more genetic and biochemical evidence in planta (Seabra and Carvalho 2015). To this end, protein immunoprecipitation coupled with mass spectrometry, which has emerged as an in vivo technology, might be helpful to solve this problem.

## PTMs in Nitrogen Signaling

### Phosphorylation of AtNRT1.1 in regulating nitrate sensing

Nitrate not only is a major source of nitrogen but also functions as an important signal molecule triggering short-term nitrate response and modulating long-term plant growth and development. In 2009, a milestone work was shown that the dual-affinity nitrate transporter, AtNRT1.1, can function as a pivotal nitrate sensor regulating an impressive range of responses to nitrate, and it is also shown that the phosphorylation state of AtNRT1.1 is responsible for the perception of external nitrate and triggering downstream primary nitrate responses (PNR) (Ho et al. 2009). Under the availability of low nitrate, AtNRT1.1 is phosphorylated at its Thr101 residue, leading to a low-level PNR; while high nitrate results in the T101 dephosphorylation of AtNRT1.1 and a high-level PNR. This phosphorylation-mediated dynamics of AtNRT1.1 is conducted by two CIPKs, CIPK8 and CIPK23, but with different regulatory modes in regulating nitrate signaling. CIPK8 acts as a positive regulator responsible for the high-level PNR, whereas CIPK23 works as a negative regulator involving in the low-level PNR by directly phosphorylating AtNRT1.1 at the Thr101 residue (Ho et al. 2009, Hu et al. 2009). Notably, CIPK23 needs to interact with the CBL1 or CBL9 (Calcineurin B-Like Protein) to form a protein complex to perform its kinase function (Ho et al. 2009). In addition, ABI2, one member of the protein phosphatase 2C family, could dephosphorylate CBL1 and CIPK23, thereby inactivating this CBL–CIPK complex and preventing AtNRT1.1 phosphorylation. Moreover, *abi2* mutant resembles the phenotype of *atnrt1.1* mutant with the loss of *AtNRT2.1* induction upon low-nitrate availability and the failure to induce lateral root elongation in the split-root experiment (Leran et al. 2015). As ABI2 is a key component involved in abscisic acid (ABA) signaling, AtNRT1.1 phosphorylation-dependent nitrate sensing and signaling may have a strong crosstalk with ABA signaling in response to different stresses.

Besides the role of AtNRT1.1 phosphorylation in the PNR, it is also involved in the long-term feedback regulation of *AtNRT2.1* expression upon high nitrate supply and lateral root development in response to differential nitrate conditions. Unlike the upregulation of *AtNRT2.1* transcripts in the PNR, *AtNRT2.1* is suppressed by AtNRT1.1-depedent nitrate signaling with long-term high nitrate supply (Remans et al. 2006). This transcriptional repression is mediated by the AtNRT1.1^T101D^ phosphomimic form but not the non-phosphorylable AtNRT1.1^T101A^ form, indicating a specific role of AtNRT1.1 phosphorylation in response to long-term high nitrate treatment (Bouguyon et al. 2015). Taken together, the phosphorylated and non-phosphorylated forms of AtNRT1.1 may have specific functions in nitrate signaling: long-term feedback regulation upon high nitrate conducted by the former and the PNR upon nitrate induction mediated by the latter. Interestingly, AtNRT1.1 also exhibits auxin transport activity and thereby regulates the elongation of lateral root by modulating its auxin transport activity dependent on the nitrate dose in Arabidopsis (Krouk et al. 2010). AtNRT1.1 represses the growth of lateral root primordia via preventing the accumulation of auxin in lateral root tips upon nitrate limitation. While nitrate is sufficient, the auxin transport activity of AtNRT1.1 is inhibited, resulting in the auxin accumulation and thus the outgrowth of lateral root (Krouk et al. 2010). Moreover, phosphorylation of Thr101 in AtNRT1.1 is necessary for regulating auxin transport activity and for the inhibition of lateral root elongation under low-nitrate availability. Expressing the phosphomimetic form NRT1.1^T101D^ in *atnrt1.1* null mutant showed a wild-type phenotype, while the transgenic line expressing the non-phosphomimic AtNRT1.1^T101A^ form in *atnrt1.1* null mutant could not resemble the phenotype of the wild type but display a similar phenotype of *atnrt1.1* null mutant with a significant increase in lateral root density and length under low-nitrate supply (Bouguyon et al. 2015). Furthermore, single-particle tracking revealed that the phosphorylable form of AtNRT1.1 has fast lateral mobility and membrane partitioning diffusion that facilitated basipetal transport of auxin to inhibit lateral root elongation under low-nitrate conditions (Zhang et al. 2019). These results indicate that phosphorylation of AtNRT1.1 is a crucial hub in the crosstalk between nitrate and auxin signaling during root development.

### PTMs in signal relays downstream of the nitrate sensor

Although AtNRT1.1 was elucidated as a nitrate receptor, how nitrate is sensed and how nitrate signal is transduced into the nucleus to alter gene expression remained elusive. Until now, two separate models of nitrate signal transduction have recently been proposed to address this problem in Arabidopsis and rice, respectively. In Arabidopsis, transcription factors AtNLP6 and AtNLP7 (NIN-like protein) were identified as the key players in the PNR (Castaings et al. 2009, Konishi and Yanagisawa 2013, Marchive et al. 2013). Interestingly, AtNLP7 is subject to cytoplasm-to-nucleus shuttling as fast as 3 min after nitrate resupply and can trigger the transcriptional alterations of a large number of nitrate-responsive genes. The cytoplasmic-nuclear shuttling of AtNLP7 reveals a fast-switch regulatory mechanism transmitting nitrate signal from the cytoplasm to the nucleus (Marchive et al. 2013). A subsequent study also revealed that subgroup III calcium-dependent protein kinases (CPK10, CPK30 and CPK32) can phosphorylate AtNLP7 at Ser205 and promote AtNLP7 retention in the nucleus, depending on nitrate-induced calcium influx (Liu et al. 2017a). By contrast, dephosphorylation of AtNLP7 is retained in the cytosol, indicating that calcium may function as a secondary messenger to regulate CPK activities and activate nitrate signaling (Riveras et al. 2015, Liu et al. 2017a). Although the nitrate-CPK-NLP module unveils the connecting of calcium signaling and nitrate-induced transcriptional response, the critical signal component connecting the nitrate sensor NRT1.1 and the master transcription factor NLP have yet to be identified.

Very recently, the study in rice proposed a different model for the mechanisms of nitrate signal propagation. It was revealed that OsNRT1.1B (the functional homologue of AtNRT1.1) can physically interact with the repressor protein OsSPX4 (the key repressor protein for phosphate signaling) and promote OsSPX4 ubiquitination and degradation in a nitrate dependent manner (Lv et al. 2014, Hu et al. 2015, Hu et al. 2019). As OsSPX4 has the ability to interact with OsNLP3 (the functional ortholog of AtNLP7) and prevent the cytoplasm-to-nucleus shuttling of OsNLP3, OsSPX4 degradation releases OsNLP3 and thereby promotes OsNLP3 shuttling into the nucleus to activate nitrate-responsive genes under nitrate stimulation. Therefore, this work unveiled an ubiquitination-mediated nitrate signaling pathway with OsSPX4 as an important hub node (Hu et al. 2019). Notably, a RING-type E3 ubiquitin ligase named NBIP1 (OsNRT1.1B Interacting Protein 1) responsible for OsSPX4 ubiquitination was also identified through immunoprecipitation followed by mass spectrometry, which underlies the molecular mechanism of OsNRT1.1B-mediated OsSPX4 degradation (Hu et al. 2019). Taken together, the OsNRT1.1B-OsSPX4-OsNLP3 cascade provides a framework for a nitrate signaling pathway propagated from the plasma membrane to the nucleus in rice.

**Fig. 3:**
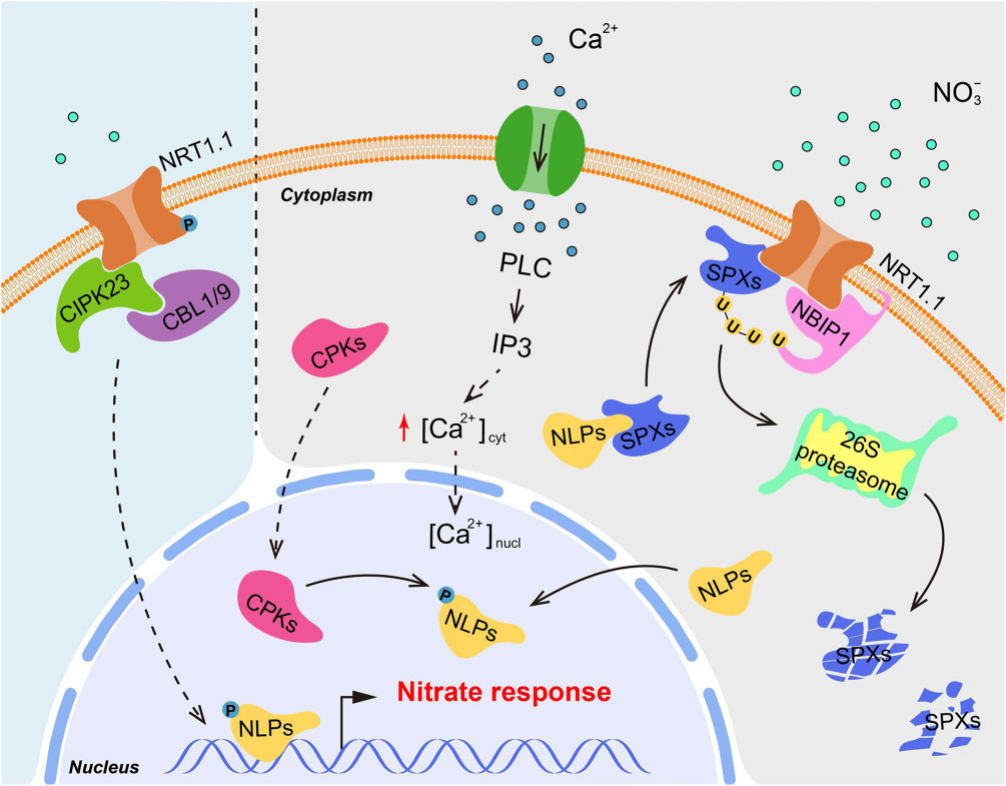
A proposed model of the nitrate signal pathway for integration of the nitrate-CPK-NLP and NRT1.1-SPX-NLP modules. Plasma membrane-localized NRT1.1 functions as a nitrate sensor in response to nitrate provision under both high and low concentrations. NRT1.1 is phosphorylated by CBL1/9-CIPK23 under low-nitrate conditions and then triggers a low-level nitrate response. In response to high nitrate provision, through Ca^2+^-dependent and Ca^2+^-independent pathway, nitrate triggers a high-level nitrate response. The import nitrate signal through NRT1.1 activates PLC activity and thereby increases the content of 1,4,5-inositol triphosphate (IP3) in the cytosol, resulting in the opening of Ca^2+^ channels and the accumulation of Ca^2+^ in the cytosol. Thus, the Ca^2+^ signal is decoded by CPKs, which in turn phosphorylate the NLPs to promote their nuclear retentions and subsequently the activation of downstream nitrate response. For the Ca^2+^-independent pathway during nitrate signal cascade amplification, NRT1.1 recruits NBIP1 to mediate the proteasome degradation of SPXs and, therefore, initiates the release of NLPs and promotes the cytoplasmic-nuclear shuttling to activate nitrate response.

In light of the repressor functions of SPX proteins and the effect of NRT1.1 involved in the nitrate-phosphate signal network in Arabidopsis and rice (Lv et al. 2014, Medici et al. 2019), it is tempting to hypothesize that the nitrate-CPK-NLP and NRT1.1-SPX-NLP modules may coexist in the same species but function at different time-points during the process of the nitrate signal cascade (Fig. 3). This hypothesis is based on the evidence that nitrate-stimulated AtNLP7 phosphorylation is triggered as fast as ten minutes, while OsSPX4 ubiquitination usually requires at least 1 h after nitrate stimulation, further suggesting that ubiquitination-mediated SPXs degradation and NLPs releasing and shuttling into the nucleus are involved in the secondary cascade amplification process of nitrate signaling. The comparative study of these two models in Arabidopsis and rice will provide a more complete framework for understanding nitrate signaling.

## Future Prospects

The biological activities of cells are not only limited by genetic code but also intensely governed by protein PTMs, which largely expand the complexity and flexibility of cellular signaling. At present, the majority of chemical modified peptides presented in plant PTM databases, including predicted by bioinformatics algorithm or identified by biochemical methods coupled with mass spectrometry, lose the context of the corresponding proteins where they were derived. Meanwhile, a single protein molecule may simultaneously undergo multiple dynamic PTMs in response to various environmental changes, which is also missing in most PTM databases (Millar et al. 2019). Nitrogen utilization and signal transduction are extremely complex and diverse to cope with nutritional changes in the environment, and multiple PTMs might concurrently participate in this process. It is believed that the interplay of PTMs provides cues to determine the fate of the proteins and consequently regulating cellular activity. However, the mechanism for crosstalk of PTMs in nitrogen utilization and signaling were still largely unknown (Vu et al. 2018, Pinto et al. 2019). The development of high-resolution mass spectrometry combined with advancement in the analysis of data generated by mass spectrometry will shed light on the functional characterization of the PTM crosstalk involved in nitrogen utilization and signaling network.

Notably, PTM identified by mass spectrometry should be coupled with detailed functional studies in plants through site-directed mutation, genetic and biochemical verification, which is quite time-consuming. Furthermore, PTMs can lead to either activation or inactivation of the specific protein as well as alterations in intracellular localization, so the functional relevance of each PTM event cannot directly be relevant to the PTM obtained by mass spectrometry. The new emerged CRISPR (clustered regularly interspaced short palindromic repeats) techniques would provide the opportunity for in situ mutagenesis to quickly characterizing the functions of PTMs detected by mass spectrometry (Zhu et al. 2020), which should be helpful in identifying key PTM sites that regulate different physiological processes in plants, including nitrogen utilization and nitrogen signaling.

## Funding

The Major Program of Guangdong Basic and Applied Research (2019B030302006) and the China Postdoctoral Science Foundation (2020M672569).
